# Deletion of Glutamate Delta 1 Receptor Leads to Heterogeneous Transcription and Synaptic Gene Alterations Across Brain Regions

**DOI:** 10.3390/ijms27010067

**Published:** 2025-12-20

**Authors:** Jingguo Huang, Jiahao Liao, Xuanying Chen, Guiping Lin, Yangwangmu De, Huakun Shangguan, Wucheng Tao

**Affiliations:** 1 Fujian Key Laboratory of Cognitive Function and Diseases, School of Basic Medical Sciences, Fujian Medical University, Fuzhou 350122, China; 2 School of Basic Medical Sciences, Fujian Medical University, Fuzhou 350122, China

**Keywords:** glutamate receptor delta 1, differential transcriptional profiling, synaptic function, ASD, ID

## Abstract

Glutamate delta 1 receptor (GluD1) has various functional roles in the brain, such as high-frequency hearing, synapse formation and maintenance, and regulation of cognition disorders and neurodevelopmental disease. However, the underlying molecular mechanism, especially at the genetic level, remains to be elucidated. In this study, we use transcriptomics analysis to define the genetic impact of GluD1 across the brain regions in GluD1 knockout mice. Our results show that GluD1 deletion induced pronounced differences in gene expression both across the four brain regions (cortex, cerebellum, hippocampus, and striatum) and the distinct hippocampal subregions. Despite differences in transcriptional profiles, the differentially expressed genes (DEGs) across all four brain regions show significant enrichment in synaptic signaling pathways, highlighting the critical role of GluD1 in synaptic function. The GluD1 interaction network and its downstream target genes are closely linked to the pathogenesis of intellectual disability (ID) and autism spectrum disorders (ASDs). In conclusion, our work reveals that GluD1 deletion leads to brain-region-specific transcriptional changes and establishes a genetic link between the interaction network with GluD1 and the risk genes for ID and ASD.

## 1. Introduction

Glutamate delta 1 receptor (GluD1), belonging to the ionotropic glutamate receptors (iGluRs), is a novel glutamate delta subunit that is encoded by *GRID1*. Identified more than two decades ago, GluD1 shares 21–25% amino acid sequence identity with iGluR subunits [1]. Although the GluD receptor was initially characterized as an atypical ion channel, a structural work on D-serine binding to GluD2 has revealed ligand-gated ion channel activity, suggesting intrinsic ligand-gating capabilities of GluD1 like GluD2 [2]. Previous studies revealed GluD1 expression in the inner ear [3] and hippocampus [4], but GluD1 has a much broader expression pattern in the forebrain [5,6,7,8]. It is widely expressed in adult mouse brains, with abundant expression in the neocortex, hippocampus, striatum, and cerebellum [5,6]. Functionally, GluD1 is crucial for excitatory synapse formation and maintenance of neural circuits [4,9,10] and controls inhibitory synapse transmission and synaptic plasticity though GABA binding [11]. Moreover, substantial evidence links GluD1 to high-frequency hearing [3], several neuronal diseases, as well as cognition disorders [12,13].

Neurological and psychiatric disorders arise from disruption in distinct neural circuits within one or more specific brain regions. Several genetic and knockdown studies have shown that GluD1 plays an important role in these diseases, such as schizophrenia [14,15,16], bipolar I disorder [14], autism spectrum disorders (ASDs) [17,18], and intellectual disability (ID) [19,20]. Based on genome-wide association studies, *GRID1* was regarded as a strong candidate for schizophrenia with shared associations between schizophrenia and bipolar I disorder though a single-nucleotide polymorphism (SNP) genotyping screen [14]. In addition, a copy number variation (CNV) study in ASD patients has discovered new candidate genes, including *GRID1*, *GRIK2*, and *GRIK4* [17]. Deletions within the 10q22-q23 genomic region, which exhibited cognitive abnormalities and ASDs, affected many known genes and putative expressed transcripts such as *GRID1* [18]. Functionally, GluD1 deletion has been associated with ASD-like behavioral deficits [21,22] and leads to deficit in reversal, contextual, and cue fear learning [12,22]. And homozygous missense variants in *GRID1* revealed that GluD1 is linked to ID and spastic paraplegia [19]. In summary, downregulation of GluD1 leads to many neurological disorders.

Given the specialized roles and signaling pathways of different brain regions, there is considerable variation in the different brain regions’ responses to GluD1. While previous studies have primarily focused on gene expression and function within a single brain region, integrative analyses comparing multiple regions remain limited, leaving cross-regional relationships insufficiently explored. Here, we use transcriptomics analysis to determine whether GluD1 deletion has distinct molecular impacts on specific brain regions. Our findings demonstrate the diverse impact of GluD1 on gene expression across the brain and provide a strong foundation for future efforts to dissect region-specific mechanisms and therapeutic strategy.

## 2. Results

### 2.1. Expression Pattern of GluD1 in the Adult Mouse Brain and Generation of GluD1 Knockout Mice

**Figure 1:**
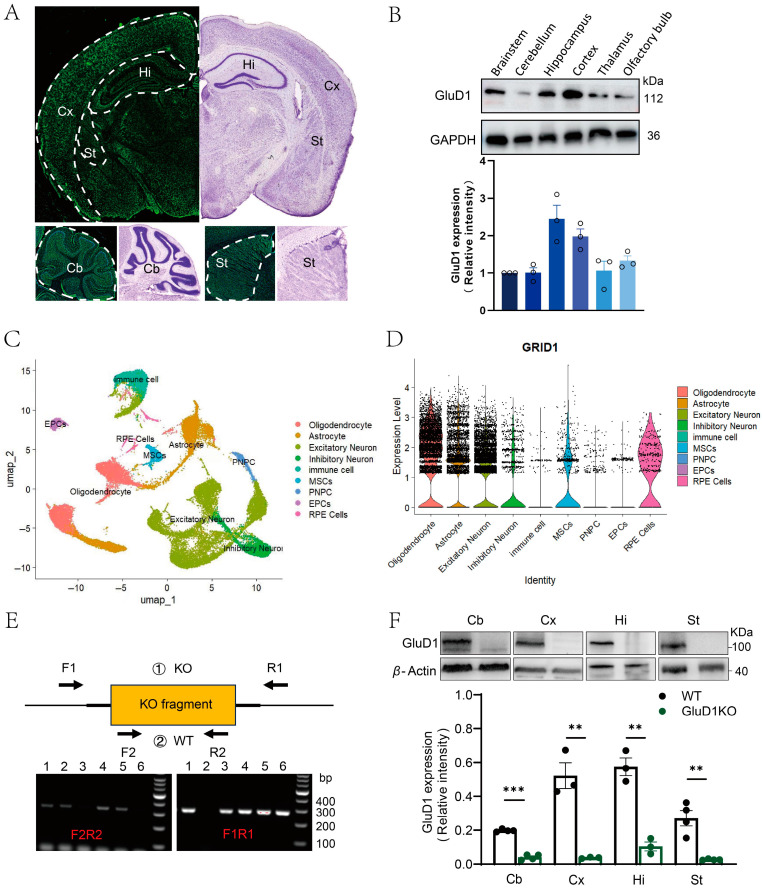
Expression pattern of GluD1 in the adult mouse brain. (**A**) Regional distribution visualized on coronal sections using immunofluorescence, revealing a broad distribution across the brain. In particular, strong immunoreactivity was detected in the cortex (Cx), hippocampus (Hi), striatum (St), and cerebellum (Cb). Schematic diagrams of the brain sections on the left are adapted from the Allen Brain Map API. (**B**) Distribution of GluD1 in different brain tissues by Western blot analysis. Quantification showed the abundance of GluD1 in different brain regions as compared to brainstem levels. GluD1 staining intensity was normalized to that of GAPDH. (**C**) Uniform manifold approximation and projection (UMAP) plot showing each brain region based on specific transcriptional signature of each spot and unsupervised clustering of these region expression profiles using a reference dataset (GSE204683). (**D**) Violin plots showing gene expression level of *GRID1* across annotated cell types. (**E**) Genotyping analysis by SDS-PAGE gel electrophoresis of GluD1 knockout mice. Lanes 1–6 represent individual mice: #3 and #6 were homozygous (−/−); #2 was wild-type (+/+); #1, #4, and #5 were heterozygous (+/−). (**F**) Expression of GluD1 in four brain regions from WT and GluD1 KO mice determined using Western blotting. GluD1 staining intensity was normalized to that of β-actin. ** *p* < 0.01, *** *p* < 0.001, two-tailed independent Student *t*-test. Quantification confirmed reduced GluD1 expression in the knockout mice.

GluD1 is expressed in adult mouse brains, such as in the hippocampus, striatum, and cerebellum [5,6]. To further characterize its expression patterns, we examined the expression levels of GluD1 in various mouse brain regions. We found high expression of GluD1 in the hippocampus, cortex, and olfactory bulb (Figure 1A). Next, we examined regional and cellular expression of GluD1 in the adult mouse brain using immunofluorescence (Figure 1B). Using a previous single-cell RNA-seq database (GSE204683) [23], we were able to identify nine distinct cell types from brains of mice. It showed the highest level in the oligodendrocyte among all CNS cell types, while the lowest of that was found in the immune cells (Figure 1C). Notably, GluD1 localized to both excitatory and inhibitory neurons (Figure 1D), consistent with previous studies [9,10]. And then we generated GluD1 knockout (KO) mice using CRISPR-Cas9 technology. GluD1 expression was examined in four brain regions in mice using Western blotting. It was shown that GluD1 expression was significantly reduced in these regions of GluD1-KO mice (Figure 1F).

### 2.2. Transcriptional Profiling in Each Brain Region of GluD1-KO Mice

**Figure 2:**
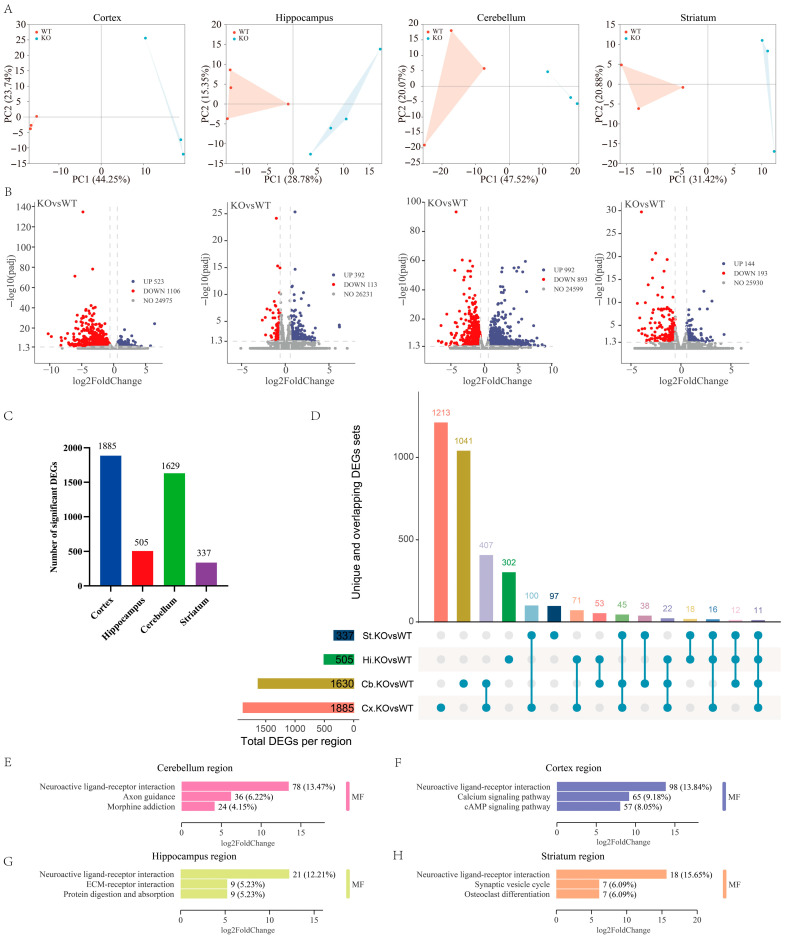
Differential transcriptional responses to GluD1 deletion across brain regions. (**A**) Principal component analysis (PCA) of transcriptome shows clustering by different regions. Each data point represents one replicate. (**B**) Volcano plots show all regulated genes for each region. The horizontal axis refers to the threshold of statistical significance with the log, while the vertical axis refers to the threshold of the differential expressed ratio. (**C**) Histogram representing the number of significant differentially expressed genes (DEGs) in four brain regions. (**D**) UpSet plot of unique genes affected in each brain region by analyzing the degree of overlap between the DEGs. The number of DEGs from each brain region is represented by the histogram on the left (0–1885 range). A single dot indicates no overlap with any other groups. Dots with connecting lines indicate one or more overlaps of DEGs between brain regions. The number of overlapping DEGs between groups is shown above each intersection. Cx: cortex, Cb: cerebellum, Hi: hippocampus, St: striatum. (**E**–**H**) Kyoto Encyclopedia of Genes and Genomes (KEGG) pathway enrichment analysis of significant DEGs based on their molecular functions (MFs) in the cerebellum (**E**), cortex (**F**), hippocampus (**G**), and striatum (**H**).

Substantial evidence indicates that GluD1 plays essential roles in synapse formation and maintenance, high-frequency hearing, and the pathophysiology of neuronal diseases. However, it remains unclear how GluD1 deletion affects transcriptomic profiling in individual brain regions. To address this question, we performed RNA sequencing (RNA-seq) on mouse brain tissues, followed by differential expression analysis (Figure 2). We examined whether local populations exhibit distinct molecular features using an unsupervised principal component analysis (PCA). The clear separation of samples from each region indicated strong region-specific transcriptional identities (Figure 2A). The volcano plots show all regulated genes for each region (Figure 2B), and the full results are provided in Supplementary Data S1. In the analysis, significant DEGs were identified using two filters, an FDR of 0.05, and an absolute fold-change threshold of 0.58. The histogram of regulated genes revealed region-dependent DEGs, with 1885 in the cortex, 505 in hippocampus, 1629 in cerebellum, and 337 in striatum (Figure 2C). The number of DEGs was highest in the cortex and lowest in the striatum. Notably, several DEGs—including *Cbln2*, *Homer*, and *Shank1*—have been previously reported as GluD1 interactors in the hippocampus [4] and in other brain regions [24], supporting the biological relevance of the detected transcriptional shifts.

We next investigated how many DEGs were uniquely affected by analyzing their cross-regional overlap (Figure 2D). Although there were many connections between different brain regions, a large proportion of DEGs (28–64%) remained region-specific. Among the identified DESs, 1213 of 1885 were unique to the cortex, 1041 of 1630 to the cerebellum, 302 of 505 to the hippocampus, and 97 of 337 to the striatum (Supplementary Data S2). Furthermore, to explore the potential function of these significant DEGs, Kyoto Encyclopedia of Genes and Genomes (KEGG) pathway enrichment analysis based on molecular functions (MFs) was performed on all brain regions, in which gene length bias was corrected. The results showed the top three enriched terms for each brain region and revealed region-specific differences in molecular function (Figure 2E–H and Supplementary Data S3). In general, the results revealed that the top pathway, neuroactive ligand–receptor interaction, was enriched in all brain regions. Beyond this common pathway, for the cerebellum, axon guidance and morphine addiction were enriched (Figure 2E). For the cortex, the calcium signaling pathway and cAMP signaling pathway were enriched (Figure 2F). The DEGs in the hippocampus were enriched for molecular functions related to ECM–receptor interaction, as well as protein digestion and absorption (Figure 2G). Finally, for the striatum, molecular functions related to synaptic vesicle cycle and osteoclast differentiation were enriched.

### 2.3. Hippocampal Subregions Exhibit Distinct Transcriptomic Signatures in GluD1 KO Mice

**Figure 3:**
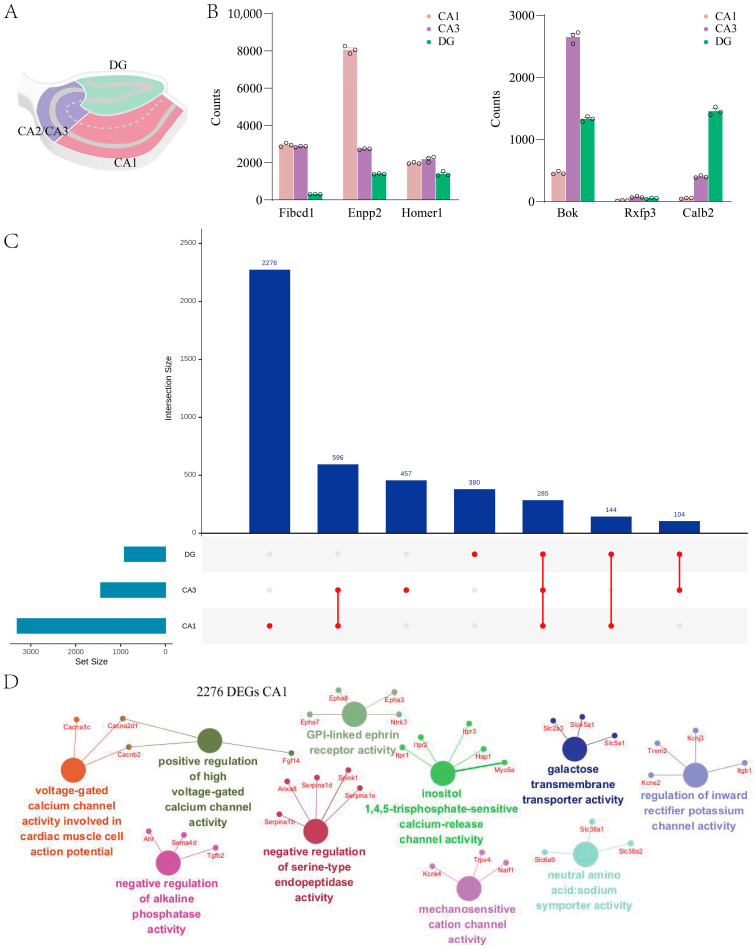
Each hippocampal subregion displayed a unique transcriptional response to GluD1 deletion. (**A**) Schematic illuminating hippocampal subregions CA1, CA2/3, and DG that were microdissected for tissue preparation. (**B**) Histogram showing region-enriched marker genes across CA1, CA2/3, and the dentate gyrus (DG). *Fibcd1* and *Enpp2* were enriched in CA1, whereas *Bok* and *Rxfp3* show elevated expression in CA3. *Calb2* displays selective expression in the DG. Each circle represents an individual biological sample. (**C**) UpSet plot of unique gene expression changes in each hippocampal subregion by analyzing the degree of overlap between the DEGs. The number of differentially expressed genes (DEGs) from each subregion is represented by the histogram on the left (0–3200 range). Genes were defined as significant using FDR step-up < 0.05 and log_2_fold-change ≥ |0.58 |. A single dot indicates no overlap with any other groups. Dots with connecting lines indicate one or more overlaps of DEGs between hippocampal subregions. The number of overlapping DEGs between groups is shown above the histogram. DG: dentate gyrus. (**D**) Molecular function (MF) enrichment analysis of DEGs unique to the hippocampal CA1 region (2276 DEGs, FDR step-up < 0.05, log_2_fold-change ≥ |0.58|).

As previous studies and our results have demonstrated, GluD1 is widely expressed in the hippocampus [6]. This brain region is mainly composed of multiple anatomical and functional substructures—CA1, CA2, CA3, the dentate gyrus (DG), and subiculum. Here we distinguished the major subregions—CA1, CA2/3, and DG—based on spatial topography (Figure 3A) as they are visually indistinguishable under a dissection microscope; CA2 and CA3 were dissected together. We confirmed the accuracy of our results through testing enrichment of known genes for each area [25,26]. These genes included *Fibcd1*, *Enpp2*, and *Homer1* in CA1; *Rxfp3* and *Bok* in CA3, and *Calb2* in the DG (Figure 3B, Supplementary Data S4). And then RNA-seq was performed in these subregions. Analysis of the transcriptomic profiles revealed unique gene expression changes and enriched molecular functions that were specific to a subregion (Figure 3C,D). Of the DEGs identified in each region, 2276/3301 DEGs were uniquely affected in CA1, 457/1442 in CA2/CA3, and 380/913 in the DG (Figure 3C, Supplementary Data S5 [25,26]).

In CA1 and CA3, several genes were enriched for postsynaptic and synaptic integration terms such as postsynaptic scaffolding proteins *Psd2* or *Homer1*, glutamate receptor subunit *Grin2a-c* or *Grin3a*, and GABA receptor subunit *Gabra1–5*, as well as acetylcholine receptor subunit *Chrna7*. Others were enriched for potassium channels such as *Kcnq1–5* and *Kcnc1–4*. For the DG, we have identified many underlying genes involved in the gene regulatory processes of mRNA, including mRNAs for transcription factors (*Xist* and *Irf7*) and Wnt signaling (*Wnt4*, *Draxin*, *Fzd9*, and *Lrp2*), which supports neurogenesis in this region. Among the regions examined, CA1 was the most affected, exhibiting the highest number of DEGs as well as the largest set of specific DEGs (Figure 3C). Enrichment network analysis was used to identify the pathways most represented among the DEGs in CA1 (Supplementary Data S6). A total of 2276 unique DEGs analyzed in CA1 revealed distinct gene expression changes and molecular functions that were uniquely enriched in CA1 (Figure 3D), which may relate to ion channel activity, such as calcium, potassium, and mechanosensitive cations.

### 2.4. GluD1 KO Neurons Exhibit Various Synapse-Related Pathways

**Figure 4:**
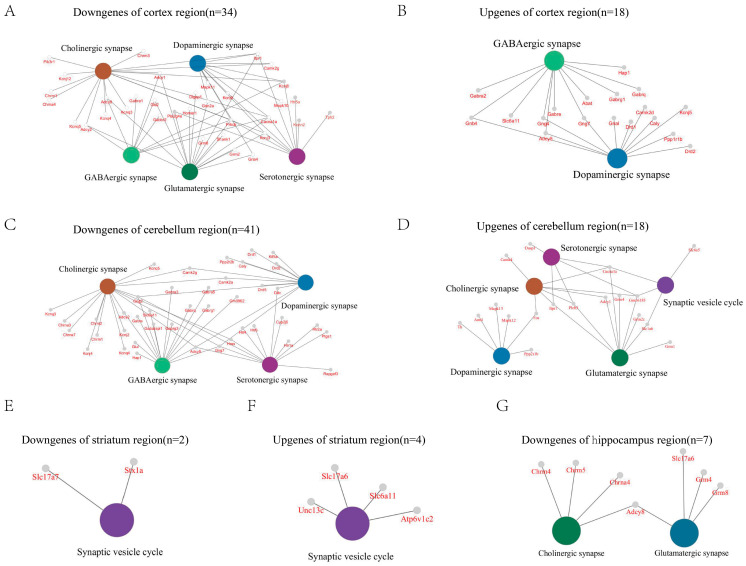
GluD1 deletion exerts region-specific effects on synapse-related genes. Gene Ontology (GO) enrichment analysis was performed on the differentially downregulated and upregulated genes based on their synaptic functions in the four brain regions. Downregulated (**A**) and upregulated genes (**B**) of cortex. Downregulated (**C**) and upregulated genes (**D**) of cerebellum. Downregulated (**E**) and upregulated genes (**F**) of striatum. Downregulated genes (**G**) of hippocampus.

To gain a better understanding of the broad transcriptomic changes mediated by GluD1 deletion, we examined DGEs which were enriched for synaptic functions. Notably, all four brain regions showed a large number of both up- and downregulated genes enriched for synaptic signaling pathways or synapse assembly processes (Figure 4). As shown in the results, the greatest number of enriched genes was found in the cerebellum (41 down, 18 up, Figure 4C,D) and the lowest in the striatum (2 down, 4 up, Figure 4E,F). In the cortex and cerebellum, DEGs were co-enriched in both excitatory and inhibitory synaptic pathways, implicating bidirectional synaptic functions of GluD1. This observation aligns with existing studies demonstrating that GluD1 plays a significant role in both excitatory and inhibitory synapses [9,10]. Dopaminergic synapses were also enriched in these two regions (Figure 4A–D). The comprehensive enrichment results are shown in Supplementary Data S7.

### 2.5. Hippocampal Proteomic Profiling Reveals GluD1 Interactors and Implicates GluD1 in ASD and ID

**Figure 5:**
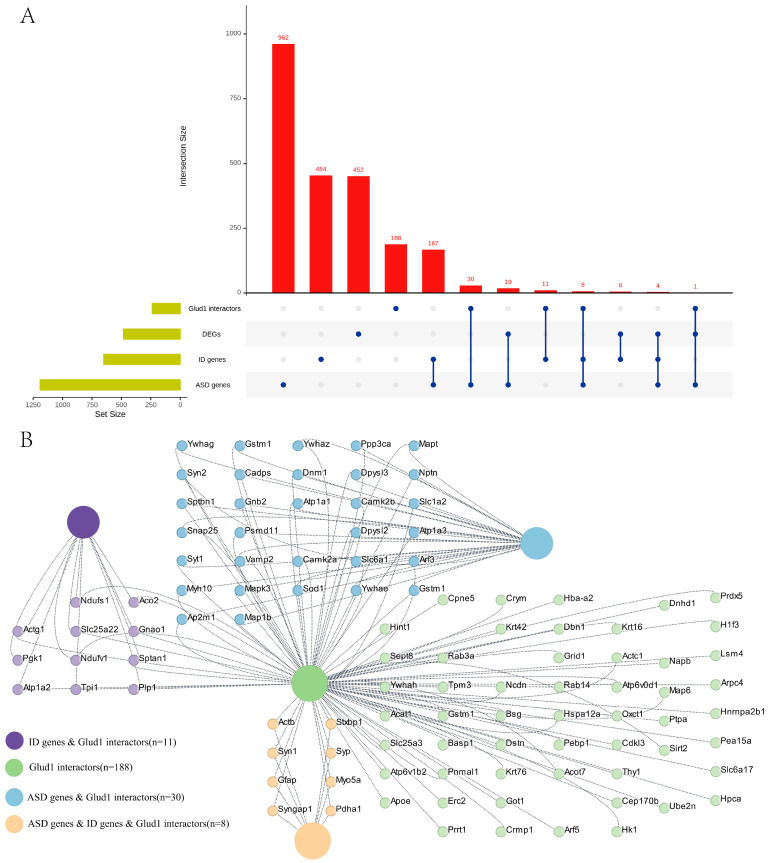
Proteomic profiling of the hippocampus identified GluD1 interactors and linked GluD1 to intellectual disability and autism spectrum disorder. (**A**) UpSet plot of interactions between GluD1 interactors, DEGs, and gene clusters associated with intellectual disability (ID) and autism spectrum disorder (ASD). The number of genes from each group is represented by the histogram on the left (0–1250 range). Genes were considered significant if FDR step-up < 0.05 and log_2_fold-change ≥ |0.58 |. A single dot indicates no overlap with any other groups. Dots with connecting lines indicate one or more overlaps of genes between groups. The number of overlapping genes between groups is shown above the histogram. (**B**) The overlapping genes are listed for the different groups.

Using proteomic analysis of mouse hippocampal tissue, we identified a set of candidate proteins that interact with GluD1. The analysis successfully delineated a robust protein–protein interaction (PPI) network (242 proteins) for GluD1 (Figure 5A, Supplementary Data S8). To investigate the role of GluD1 in disease regulation, we performed an integrative analysis using ASD- and ID-related gene databases [27,28]. A total of 1191 ASD-associated genes and 650 ID-associated genes were obtained from the databases, respectively. These gene clusters were then integrated with the DEGs and the GluD1 interactors to identify overlapping genes (Figure 5A). The results showed that a considerable portion of the target genes overlapped across the four clusters. Among them, the greatest overlapping was observed between the GluD1 interactors and risk factors for ASD, highlighting the critical role of GluD1 in ASD [17,18,21]. Notably, four genes (*Aspm*, *cdk5rap2*, *dmpk*, and *Satb2*) were detected in the DEGs, ID genes, and ASD factors (Figure 5B). We also identified genes related to cytoskeletal proteins (*Actb*, *Gfap*, and *Myo5a*), energy metabolism (*Pdha1*), and synaptic function (*Stxbp1*, *Syn1*, *Syngap1*, and *Syp*) within the overlap of the GluD1 interactors and risk factors for both disorders (Figure 5B). The findings are in agreement with our GO enrichment analysis (Figure 4).

## 3. Discussion

In this study, we uncover a comprehensive transcriptomic signature across multiple brain regions. Our results show that GluD1 deletion causes region-specific alterations in gene expression and disrupts synaptic signaling pathways, with the most pronounced effects observed in the hippocampal CA1. Furthermore, we identify a significant overlap among the genes coded by the GluD1 interactors, DEGs, and genetic risk factors for ASD and ID. Together, these results highlight an important role for GluD1 in neuronal functions and may provide new insights into the therapeutic strategies for neurodevelopmental disorders.

GluD1 was transcribed weakly in the adult brain regions comparing to other excitatory receptors [29]. In comparison, higher-sensitivity mRNA analysis revealed that widespread and robust neuronal expression of GluD1, including in the hippocampus and cerebellar cortex [5,6]. Consistent with these findings, our data show that GluD1 is expressed in four brain regions in the adult mouse. In addition, the highest number of DEGs is found in the cortex (Figure 2A), indicating that GluD1 may contribute to the major regulatory effect on the cortex. This is in line with the fact that the cerebral cortex is an extensive structure comprising various functional regions and exhibits high levels of GluD1 expression [6,30,31].

We further analyzed the degree of overlap among DEGs to investigate how many genes are uniquely affected in each brain region. Notably, this conservative approach highlights the heterogeneity of brain region response to GluD1 as we identified only 11 overlapping DEGs across the four regions (Figure 2B). A potential explanation for this is that each brain region carries out distinct major functions. The cortex, cerebellum, and striatum exhibit substantial overlap in their DEGs, which likely reflects their common roles in functions, such as the regulation of motor activity [32,33,34,35]. Our findings also demonstrate that heterogeneous transcriptomic signatures in distinct hippocampal subregions respond to GluD1 deletion. These subregions differ by their neural circuitry, cell types, and electrophysiological characteristics. Whereas previous studies have primarily focused on gene expression and function in the hippocampus as a whole, our study provides a comprehensive view of transcriptomic alterations across subregions. Notably, the majority of DEGs in the individual subregions are unique, but a considerable portion of them are common to all. These genes are predominantly related to postsynaptic and synaptic function, ion channels, axonal guidance, and neuronal recognition. In general, our study illuminates the transcriptomic diversity that exists across brain regions and hippocampal subregions and helps define a structure-related functional map.

Increasing evidence has showed considerable diversity in the structure, molecular composition, and function of synapses [36,37,38]. In this study, we assessed regional heterogeneity by examining the enrichment patterns of synapse-related DEGs in the four brain regions. This analysis revealed distinct region-specific synaptic signatures, highlighting the complexity and diversity of synaptic functions across brain regions. Pathways related to glutamatergic-, GABAergic-, cholinergic-, dopaminergic-, and serotonergic-synapses are significantly enriched in the cortex and cerebellum. Other studies have reported that GluD1 acted as the key role in synaptic functions, consistent with our findings. For example, deletion of GluD1 decreased excitatory synaptic transmission in the VTA, hippocampal CA1, and Dorsal raphe [4,39,40] while increasing overall excitatory transmission in the prelimbic cortex, striatum, and subiculum [10,41,42]. Several reports have also showed decreased inhibitory synaptic transmission across multiple cortical regions and the NAc core following GluD1 deletion [9,13,43]. Moreover, a causal link was established between *GRID1* gene alteration and dopaminergic dysfunction in DA neurons of the midbrain [39]. And the function of GluD1 on synaptic plasticity has been thoroughly researched [3,11,44]. Although fewer synapse-related genes are detected in the hippocampus and striatum, substantial research has already elucidated their function [4,10,42,44]. This suggests the inherent limitations of the transcriptomics technique, which may not fully capture functional synaptic changes. Nonetheless, transcriptomic data remains highly informative, especially when integrated with complementary approaches like proteomics.

Finally, we observed no overlap between GluD1 interactors and the DEGs. The lack of overlap may arise from both biological and technical factors. Proteomic analysis measures protein abundance and stability, which do not always correlate with mRNA expression. In addition, the proteomic data was exploratory, lacked biological replicates, and may have limited sensitivity for low-abundance proteins. Differences in brain region, cell type, or developmental timing between assays could further reduce concordance. Therefore, we interpret the proteomics-based interactions as hypothesis-generating and emphasize the need for targeted experimental validation in future studies. To further strengthen our study, we performed an integrative analysis combining publicly available ASD- and ID-related datasets with our own results. The significant overlap among these clusters strongly positions the GluD1 interactors as a pivotal hub in the pathophysiology of neurodevelopmental disorders. Importantly, our PPI data is derived from the hippocampus, a region critically involved in learning and memory, which are frequently impaired in ID and a subset of ASD [45,46,47,48]. This suggests that disruption of the GluD1 interactors in hippocampal circuits may directly lead to cognitive and memory deficits. Collectively, our results integrate existing functional evidence with our new proteomic and transcriptomic data, implying that GluD1 may be involved in molecular mechanisms underlying ID and ASD.

A limitation of the present study is that we did not perform a direct gene-level comparison with public RNA-seq datasets. We searched major repositories but did not identify publicly available datasets matching our experimental context. Future work will include direct comparisons when suitably matched datasets become available.

In conclusion, our study delineates the region-specific transcriptomic profiles of GluD1 deficiency and reveals marked heterogeneity across both brain regions and hippocampal subregions. By identifying distinct synaptic signaling disruptions, we highlight the selective vulnerability of specific circuits to GluD1 loss. Furthermore, through integrative analysis, we show that genes within the GluD1 interactors or DEGs converge with genetic risk factors for ID and ASD. These findings position GluD1 as a mechanistic node linking synaptic dysfunction to neurodevelopmental mechanisms, providing a strategic foundation for future therapeutic targets.

## 4. Materials and Methods

### 4.1. Animals

All mice were kept in individual ventilated cages in a 12 h light/12 h dark cycle with free access to food and water, and the room temperature was maintained at around 24 °C. Wild-type (WT) C57BL/6 mice were obtained from the laboratory animal center of Fujian Medical university. GluD1 knockout mice were constructed by the company GemPharmatech Co., Ltd., Nanjing, China. All the experimental procedures on animals were approved by the Institutional Animal Care and Use Committee of Fujian Medical University.

### 4.2. Immunofluorescence

Mice were intracardially perfused with PBS. After post-fixation with 4% paraformaldehyde (PFA), dissected brains were dehydrated in 20% sucrose solution for 24 h and in 30% sucrose solution for at least 48 h then embedded with optimal cutting temperature compound (OTC) at −80 °C. Sagittal sections measuring 10 μm were obtained using a cryostat microtome and further used for immunofluorescence. In brief, slices were incubated in 0.1 M sodium citrate solution at 97 °C for 30 min. Slices were washed with PBS three times for 10 min. Next, 0.3% Triton X−100, 3% normal goat serum (Jackson ImmunoResearch Laboratories, West Grove, PA, USA) and 2% BSA (Sigma-Aldrich, Darmstadt, Germany) in PBS were used to block unspecific staining. And then, primary antibodies (rabbit anti-GluD1, 1:200, Frontier Institute, Hokkaido, Japan) were incubated for 48 h at 4 °C and secondary antibodies (AIexa Fluor 488, 1:1000, Invitrogen, Carlsbad, CA, USA) for 2 h at room temperature. Both primary and secondary antibodies were diluted in blocking solution. Finally, antifade mounting medium (with DAPI, servicebio, Wuhan, China) was added. Images were obtained by THUNDER Imaging Systems (Leica, Wetzlar, Germany).

### 4.3. Western Blotting

The tissue samples from the striatum, hippocampus, cortex, and cerebellum were lysed using radioimmunoprecipitation assay buffer (RIPA buffer) containing protease and phosphatase inhibitor cocktails (Thermo Fisher Scientific, Waltham, MA, USA). Protein samples were separated by 8% SDS-PAGE under reducing conditions and electrophoretically transferred to PVDF membranes (Millipore, Merck, Darmstadt, Germany). Membranes were blocked with 5% skimmed milk for 2 h and incubated with primary antibody (rabbit anti-GluD1, 1:1000; mouse anti-β-actin, 1:10,000; mouse anti-GAPDH, 1:10,000) overnight at 4 °C and HRP-conjugated secondary antibodies (1:10,000, Jackson Immunoresearch, West Grove, PA, USA) at room temperature for 1 h. Protein bands were visualized with an enhanced chemiluminescence (ECL) kit (Immobilon, Merck, Darmstadt, Germany). Intensity values for GluD1 were normalized to β-actin or GAPDH.

### 4.4. Tissue Processing and Transcriptome Sequencing

The tissues from each brain region were dissected from 3 WT and 3 GluD1-KO mice at the age of 8 weeks. And hippocampal subregions were obtained as described previously [49]. The samples extracted above were used for RNA-seq. Total RNA was extracted by using TRIzol and assessed using the RNA Nano 6000 Assay Kit and the Agilent Bioanalyzer 2100 system (Agilent Technologies, Santa Clara, CA, USA). The library was checked with Qubit and real-time PCR for quantification and bioanalyzer for size distribution detection. The fragmented cDNA pools were sequenced using an Illumina NovaSeq 6000 system (Novogene Corp., Sacramento, CA, USA) with a target of 200 million total read pairs. Raw reads were subjected to quality control using FastQC (v0.11.9), and multi-sample QC summaries were generated with MultiQC (v1.14). All the downstream analyses were based on the clean data with high quality. Processed reads were aligned to the mouse reference genome (GRCm39/mm39). An index of the reference genome was built, and paired-end clean reads were aligned to the reference genome using Hisat2 (v2.0.5). FeatureCounts (v1.5.0-p3) was used to count the read numbers mapped to each gene. And then FPKM of each gene was calculated based on the length of the gene and read count mapped to this gene. DEGs between the two groups were identified using the R software ‘DESeq2’ R package (v1.20.0). The Benjamin–Hochberg method was applied to control the false discovery rate (FDR). The significant DEGs were identified using two filters, an FDR of 0.05, and an absolute fold-change threshold of 0.58.

Principal component analysis (PCA) and volcano plots were generated with ggplot2 (v3.4.4) and EnhancedVolcano (v1.18.0). KEGG pathway enrichment analysis for DEGs was conducted with the clusterProfiler R package (v3.8.1) via the enrichKEGG() function within an over-representation analysis (ORA) framework. To minimize potential gene length bias, gene identifiers were de-duplicated before enrichment testing (one gene, one entry), and a consistent background gene universe matched to the differential expression analysis was applied to avoid inflated pathway contributions driven by transcript-length redundancy or read-coverage dependence.

### 4.5. Shotgun Proteomics

The identification and quantification of multiple proteins were performed using shotgun proteomics. Proteins obtained from the Co-Immunoprecipitation (Co-IP) assay were first subjected to enzymatic digestion, and the resulting peptides were separated by liquid chromatography (LC) and analyzed by tandem mass spectrometry (MS/MS) to achieve peptide and protein identification.

### 4.6. Co-Immunoprecipitation Assay and SDS-PAGE

The co-IP assay was conducted according to a previous study with minor modification [50]. In brief, mouse hippocampal tissues were incubated with ice-cold RIPA buffer (NCM Biotech, Suzhou, China) containing protease inhibitor cocktail (Beyotime Biotechnology, Shanghai, China) for 15 min on ice. The mix solution was centrifugated at 13,000× *g* for 10 min at 4 °C, and the supernatant was transferred to a microcentrifuge tube. Normal rat polyclonal IgG and Protein A/G-Agarose (25%, *v*/*v*) were added to the tube and incubated at 4 °C for 1 h on a rotator. The solution was centrifuged at 1000× *g* for 5 min at 4 °C and transferred to a new microcentrifuge tube. The primary antibody (rabbit anti-GluD1, 1:1000, Frontier Institute) was added and incubated at 4 °C for 2 h. The suspended Protein A/G-Agarose (25%, *v*/*v*) was added and incubated at 4 °C overnight. The immunoprecipitates were collected by centrifugation at 1000× *g* for 5 min at 4 °C and gently washed three times using ice-cold RIPA buffer containing protease inhibitor cocktail. The pellet was then resuspended with 2× electrophoresis loading buffer (Thermo Fisher Scientific, Waltham, MA, USA). Finally, the immunoprecipitated samples were boiled for 3 min and stored at −20 °C for use. The immunoprecipitated samples were separated by sodium dodecylsulfate polyacrylamide gel electrophoresis (SDS-PAGE) and stained for total protein with Coomassie.

### 4.7. Liquid Chromatography and Mass Spectrometry Analysis

The gels were sent to GeneChem (Shanghai, China), and the assays were conducted on a Q Exactive mass spectrometer coupled with an Easy nLC (Thermo Fisher Scientific, Waltham, MA, USA). Briefly, the whole peptide sample was extracted and digested from the gel sample stained with Coomassie blue. The 200 ng (20 ng/μL) trypsin was added to digest overnight at 37 °C. And then the sample was separated on the C18 reversed phase analytical column (Thermo scientific, Acclaim PepMap RSLC 50 um × 15 cm, nano viper). The Easy nLC system was used to deliver different solutions at a flow rate of 300 nl/min. Buffer A comprised 0.1% formic acid in HPLC grade water, and buffer B consisted of 80% acetonitrile and 0.1% formic acid. The linear chromatographic gradient was achieved with a linear increase in buffer B percentage, which was set up as follows: 6% buffer B for 5 min, 6–28% buffer B for 40 min, 28–38% buffer B for 5 min, 38–100% buffer B for 5 min, and hold in 100% buffer B for 5 min. After that, the peptide was analyzed by the Q Exactive mass spectrometer. MS data was acquired by a data-dependent top 10 method dynamically choosing the most abundant precursor ions from the full scan (350–1800 *m*/*z*) for HCD fragmentation.

### 4.8. Protein Identification and Annotation

Raw MS data were processed in Proteome Discoverer 2.2 (Thermo Fisher Scientific) for protein identification and searched using the Mascot 2.6 engines (Matrix Science, London, UK). The following parameters were used for protein identification: trypsin as the digestion enzyme, up to 2 missed cleavages, precursor mass tolerance ± 10 ppm, fragment mass tolerance 0.05 Da, carbamidomethyl (C) as a fixed modification, oxidation (M) and protein *n*-terminal acetylation as variable modification, and target-decoy FDR control with peptide FDR ≤ 0.01. Interacting proteins were annotated as described in the main text.

### 4.9. Single-Cell Transcriptome Analysis

Single-cell RNA-seq results were obtained by reanalyzing a publicly available dataset (GSE204683). Briefly, the raw count matrix and associated cell metadata were downloaded and analyzed using Seurat. Quality control filtering excluded low-quality cells based on the percentage of mitochondrial transcripts, the number of detected genes per cell, and total UMI counts. After filtering, the dataset retained cells with an average of 1500 detected genes. Data were normalized and variance-stabilized using standard Seurat workflows, followed by scaling and dimensionality reduction. Clustering was performed based on highly variable genes, and violin plots were generated to visualize *GRID1* expression across annotated cell subpopulations.

### 4.10. Statistics and Reproducibility

All experiments in this study were repeated at least three times. GraphPad Prism, version 8.0 software, was used for all statistical analysis. The experimental data presented in all figures are expressed as the mean  ±  standard error (SE). The Mann–Whitney U test was used for intergroup comparison, and *p* < 0.05 was considered statistically significant.

## Data Availability

The original contributions presented in this study are included in the article. Further inquiries can be directed to the corresponding authors.

## Supplementary Materials

The following supporting information can be downloaded at: https://www.mdpi.com/article/10.3390/ijms27010067/s1.

## Abbreviations

GluD1	glutamate delta 1 receptor
iGluRs	ionotropic glutamate receptors
ASD	autism spectrum disorder
ID	intellectual disability
SNP	single-nucleotide polymorphism
CNV	copy number variation
KO	knockout
DEGs	differentially expressed genes
KEGG	Kyoto Encyclopedia of Genes and Genomes
MF	molecular function
RNA-seq	RNA sequencing
GO	Gene Ontology
PPI	protein–protein interaction
QC	quality control
FPKM	fragments per kilobase of exon model per million mapped fragments
ORA	over-representation analysis
FDR	false discovery rate
PCA	principal component analysis
